# The Effect of Coronavirus Outbreak on the Utilization of Coronary Revascularization Procedures: An Interrupted Time Series Analysis

**DOI:** 10.3390/jcdd10030102

**Published:** 2023-02-27

**Authors:** Antonio Sarria-Santamera, Alexandr Petrov, Dinara Yessimova, Miguel A Ortega, Saule Zhumambayeva, Angel Asúnsolo

**Affiliations:** 1 Department of Biomedical Sciences, Nazarbayev University School of Medicine, Nur-Sultan 010000, Kazakhstan; 2 Department of Medicine and Medical Specialties, Faculty of Medicine and Health Sciences, University of Alcalá, 28801 Alcala de Henares, Spain; 3 Ramón y Cajal Institute of Sanitary Research (IRYCIS), 28034 Madrid, Spain; 4 Department of Children Disease, Astana Medical University, Beibitshilik St 49/A, Astana 010000, Kazakhstan; 5 Department of Surgery, Medical and Social Sciences, Faculty of Medicine and Health Sciences, University of Alcalá, 28801 Alcala de Henares, Spain

**Keywords:** coronavirus disease 19 (COVID-19) pandemic, interrupted time series (ITS) analysis, coronary revascularization, percutaneous coronary intervention (PCI), cardiac artery bypass graft (CABG)

## Abstract

The coronavirus disease 19 (COVID-19) pandemic represented a great challenge for health systems, which had to quickly readapt and dedicate most of their resources to managing this crisis. The postponement of programmed interventions such as coronary revascularization procedures represented a critical issue in the first wave of the COVID-19 pandemic, especially in the hardest-hit countries such as Spain. However, the precise consequences of the delay of coronary revascularizations are not clearly determined. In the present work, interrupted time series (ITS) analysis was used to evaluate the utilization rates and assessment of the risk profiles of patients receiving two main coronary revascularization procedures (percutaneous coronary intervention—PCI and coronary artery bypass graft—CABG) and compared them in the periods before and after March 2020 using the Spanish National Hospital Discharge Database (SNHDD). Our results show that the abrupt reorganization of hospital care that represented the first wave of COVID-19 in March 2020 in Spain led to a reduction in cases, which was accompanied by an increase in the risk profile of CABG patients, but not PCI. On the other hand, the risk profile of both coronary revascularization procedures began before the pandemic, showing a significant temporal trend toward an increase in the risk profile. Future works should be directed to study and validate our results, evaluating other databases, regions, or countries.

## 1. Introduction

Spain was one of the countries more severely affected by the first COVID-19 wave [1]. To accommodate the unprecedented stress to treat the unexpected and extraordinarily high number of patients during the first wave in March 2020, hospitals had to quickly re-organize, dedicating almost all hospital resources to treating patients with SARS-CoV-2 and canceling most programmed admissions. The immediate consequence was a reduction even in cases requiring emergency interventions [2]. Prioritization among patients with heart diseases requiring coronary revascularizations has represented a difficult issue during the COVID-19 pandemic [3]. After that first wave, hospitals established new COVID-19-negative units for the care of patients requiring non-COVID-19 related care, including coronary revascularization for the delivery of safe and effective care even in the epicenter of the pandemic. The late effects of the postponement of coronary revascularizations are not clearly determined [4,5].

Percutaneous coronary intervention (PCI) and coronary artery bypass grafts (CABG) are the two main techniques used for revascularization procedures for patients with chronic coronary artery disease. Both procedures have been showing different trends in their utilization in Spain in recent years: on the one hand, in the last two decades, PCI showed a three times increase, whereas, on the other hand, the proportions of CABG reduced by 27% [6].

Interrupted time series (ITS) analysis has been proposed as a robust design to evaluate the impact of interventions when their implementation occurs at a concrete moment in time [7,8]. For this, it is necessary to have the observed data of the variable of interest during a period (time series) that includes measurements both before and after the application of the intervention, which is the moment of interruption of the series. The effects of the intervention are assessed by changes in the level and slope of the time series, as well as by the statistical significance of the different model parameters.

Given the uncontrolled COVID-19 epidemiological situation, on 14 March 2020, the government approved declaring a state of alarm throughout the Spanish territory to deal with the health emergency caused by COVID-19. The state of alarm was extended until 00:00 on 21 June 2020 [9]. After the first period of the pandemic, hospitals were returning to usual care.

This work aims to explore the effect that the COVID-19 pandemic had on the use of revascularization procedures in Spain, both in utilization rates and also assessing the risk profiles of patients receiving both PCI and CABG, comparing the periods before and after March 2020, when the Spanish government introduced the state of alarm.

## 2. Materials and Methods

### 2.1. Study Design and Population

We conducted a descriptive epidemiological study using the SNHDD. The SNHDD is an administrative database managed by the Spanish Ministry of Health (SMH) that collects information from all private and public hospitals. These hospitals are required by law to provide data from all subjects hospitalized for at least 24 h. The following variables for each patient are included in the SNHDD: age, sex, place of residence, dates of admission and discharge, discharge destination, primary diagnosis, secondary diagnosis (up to a maximum of 19), and procedures (therapeutic or diagnostic) conducted during the hospitalization period (up to a maximum of 20). The SNHDD applies the International Classification of Disease Tenth Revision (ICD10) to codify diagnoses and procedures. Details on the SNHDD can be found online [10]. In this work, we evaluated patients registered in the Spanish National Hospital Discharge Database (SNHDD) with a code indicating they underwent PCI or CABG during the 5-year period, from January 2016 to December 2020.

### 2.2. Statistical Analysis

An ITS analysis was performed with the data. First, a descriptive analysis of the data was conducted. The risk profile of each individual patient is based on the predicted probability of death in patients who underwent CABG and PCI. The analysis was conducted on two cohorts: patients who underwent PCI and CABG separately. The risk profile was determined by estimating the probability of death for each patient obtained through a logistic regression model, using the patient discharge status (dead or alive) as an outcome variable. The variables included in those models to estimate the risk of death were age, sex, admission to ICU, severity, mortality risk, and Elixhauser Comorbidity Index. The Elixhauser Comorbidity Index is a system for classifying a patient’s comorbidities using ICD diagnosis codes from administrative data sources, such as hospital abstracts. The categories are binary and indicate the presence or absence of a comorbidity. The index can be used to anticipate hospital resource utilization and in-hospital death rates [11]. This statistical analysis was performed via IBM SPSS Statistics, version 26.

**Figure 1:**
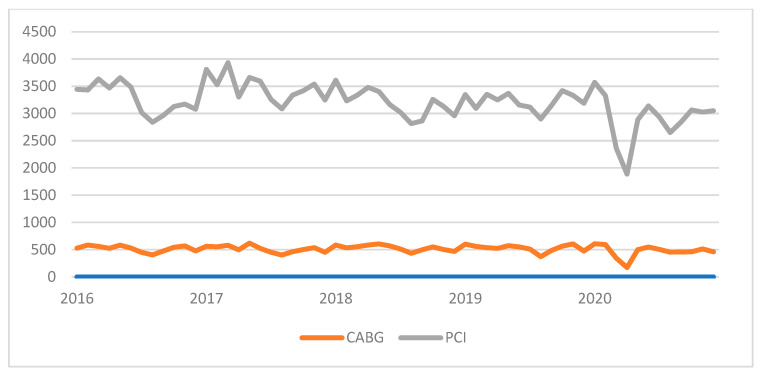
Time trends of coronary revascularizations in Spain.

The time intervals were 60 months in a five-year period from 2016 to 2020. Segmented regression analysis was used as a method of modeling the time series. First, time series were checked for seasonality and were non-stationary. The patterns of each are clearly seen on the decomposition plots in Figure 1. The seasonality of the series was controlled using the “seasonal” adjustment function in RStudio during the plot construction. However, an ordinary least squares function with the addition of dummy variables for months from January to November was used to adjust the model for seasonality in order to determine the coefficients. The general time series model was as follows:

Deathprob_t_ = β_0_ + β_1_⋅t + β_2_⋅emergency_t_ + β_3_⋅emergency_t_ t + ε_t_

where t refers to month t;

deathprob_t_ is the estimated probability of death;

emergency_t_ is a dummy variable equal to 0 until March 2020, but equal to 1 from April 2020 onward;

β_0_ is the intercept and corresponds to the death probability in January 2016;

β_1_ is the slope of the trend before the intervention;

β_2_ is the change in the level caused by the intervention;

β_3_ is the change in the trend of the slope, caused by the intervention;

The interrupted time series analysis was conducted in the RStudio 2022.07.1 + 554.

## 3. Results

**Figure 2:**
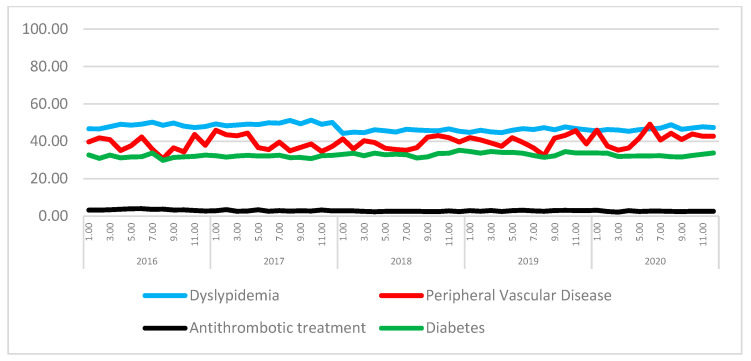
Time trends of relevant factors and treatments in patients with coronary revascularizations.

Data from 306,910 patients who underwent PCI or CABG during the 5-year period from January 2016 to December 2020 were included in this analysis. The overall PCI and CABG procedures analyzed in this study are shown in Figure 1. The main characteristics of the patients receiving either procedure are shown in the Supplementary Table. Figure 2 shows the time trend of significant coronary factors, including the antithrombotic treatment of patients with coronary revascularizations.

The logistic regression model to determine the risk profile for PCI was overall statistically significant, ($\chi^{2}$ (8) = 17,520.740, *p* < 0.001), explained with 42.3% (Nagelkerke R^2^), and correctly predicted 97.5% of cases. Age (*p* < 0.001), gender (*p* < 0.001), severity (*p* < 0.001), risk mortality, and Elixhauser comorbidity index were all significant in the predicted probability of death in PCI patients.

Turning to the predicted probability of death in CABG patients, the model was overall statistically significant, ($\chi^{2}$ (8) = 3180.226, *p* < 0.001), explained with 32.3% (Nagelkerke R^2^), and correctly predicted 95.6% of cases. Age (*p* < 0.001), gender (*p* < 0.001), severity (*p* < 0.001), risk mortality, and Elixhauser Comorbidity Index were all significant but admission to ICU (*p* = 0.116) was not.

**Figure 3:**
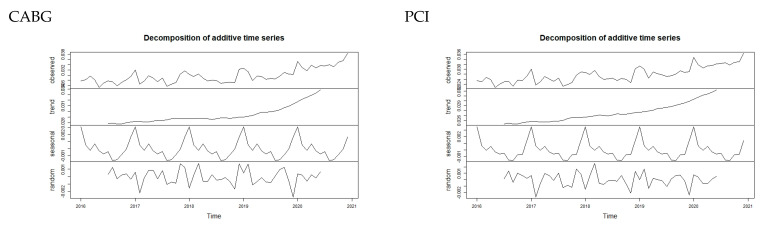
Decomposition of additive time series.

It was found that time series had seasonality and were non-stationary. The patterns of each are clearly seen on the decomposition plots in Figure 3.

General models of segmented regression equations:

CABG: Outcome = 2.890 × 10-02 + 6.870 × 10^−5^⋅time + 2.512 × 10^−3^⋅level + 2.983 × 10^−4^⋅trend + ε_t_

PCI: Outcome = 2.569 × 10-02 + 1.072 × 10^−4^⋅time + 1.747 × 10^−3^⋅level + 2.527 × 10^−4^⋅trend + ε_t_

**Figure 4:**
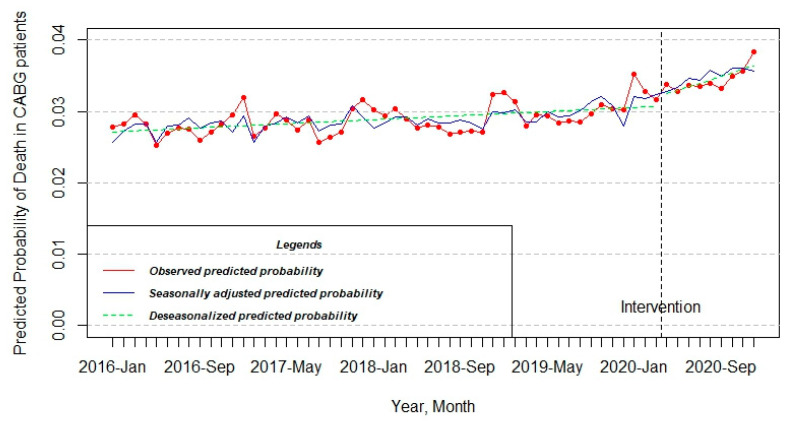
Interrupted time series model of the change of predicted probability of death in patients who underwent CABG.

It is clearly seen graphically from Figure 4, that the predicted probability of death among the CABG patients rose slightly during the period of five years from January 2016 to December 2020, from 0.028 to 0.038, respectively. After adjusting for seasonality, the model shows that there was a statistically significant change in level by 2.512 × 10^−3^ after the introduction of the lockdown (estimate = 2.512 × 10^−3^; Std. error = 1.056 × 10^−3^; t value = 2.379; Pr(>|t|) = 0.02164) (Table 1).

The joint hypothesis test robust to autocorrelation and heteroskedasticity rejected the null hypothesis for both CABG (4.204 × 10^−7^) and PCI (3.531 × 10^−7^) (Table 2). Thus, dummy variables in the unrestricted models statistically significantly enhance the general fit of the model.

**Figure 5:**
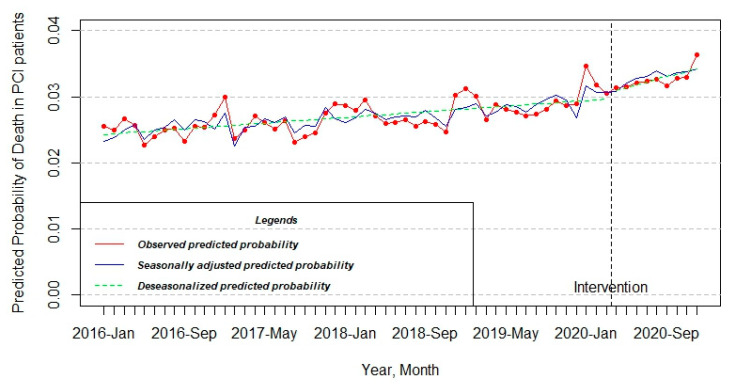
Interrupted time series model of the change of predicted probability of death in patients who underwent PCI.

The pattern of the PCI model also experienced a little increase in the observed predicted probability throughout half a decade from 0.025 to 0.036. On the other hand, the change at that level was not statistically significant after the approval of intervention (estimate = 1.747 × 10^−3^; Std. error = 1.089 × 10^−3^; t value = 1.604; Pr(>|t|) = 0.115772) (Table 1 and Figure 5).

## 4. Discussion

The main finding of this work is that the outbreak of COVID-19 in Spain did have an initial significant reduction in the volume of coronary revascularizations but did not influence the risk profile of patients admitted for PCI, and it showed a small, although significant, level effect in the risk profile of CABG in Spanish hospitals.

With the first wave of COVID-19 cases, hospitals were forced to abruptly reduce admissions for the care of non-COVID-19 patients, including patients with chronic ischemic disease [12], but our data suggest that the reduction was not associated with lower severity of cases for PCI, whereas it showed an increase for patients admitted for surgical revascularizations associated with the abrupt COVID-19 hospital reorganizations.

The results from ITS models reveal, interestingly, that already before the March 2020 surge in COVID-19 cases and the introduction of lockdowns and state of alarm in Spain, there was an increase in patients’ risk profiles receiving either PCI or CABG. The significant time effect observed reflects that cardiologists’ and cardiac surgeons’ decision-making regarding indications for both revascularization procedures were already selecting more severe at-risk patients.

What is also quite an interesting finding is the seasonality of time series in both the PCI and CABG models. Specifically, our study uncovers seasonality in the risk profiles of treated patients. A study by Stewart et al. (2017) supports our finding by mentioning that the majority of studies state higher hospitalization and mortality frequency during the winter months in comparison to summer periods. They also suggest that the reason behind this issue is multifactorial and may be related to physiological responses to cold and heat, behavioral changes in particular seasons, vitamin D deficiency, ambient air pollutants, and susceptibility to infectious diseases [13].

ITS represents a valuable study design to assess the effect of population-based interventions when randomization is not possible or feasible. Natural experiments could be considered for evaluating health or other outcomes where exposure to the event of the intention of interest has not been manipulated by the researcher [14]. Natural experiments require clever approaches to analyzing data, such as comparing what happened before and after lockdowns using methods that could include interrupted time series, or between a population that was subject to lockdown and another that was not, which can serve as a control [15].

The pandemic has created a natural experiment of unprecedented proportions. The COVID-19 pandemic has resulted in considerable delays and affected the patients’ clinical profiles due to the implementation of restrictive measures in cardiac revascularization units [16], but it also provided this opportunity to assess the dynamics of the risk profile of patients receiving coronary revascularizations.

A common finding observed during the COVID-19 pandemic is a reduction in revascularization volumes, whereas some authors have found higher risk-adjusted mortality [17] and others have not [18]. This study did not aim to explore the mortality of patients and has some limitations. The most important is that the post-COVID-19 period includes only nine observations. Another relevant limitation is the capacity of administrative data to capture appropriate clinical variables reflecting the severity of chronic coronary patients. Additionally, this study analyzes national data, and the observed effects may have regional or local differences.

## 5. Conclusions

The abrupt reorganization of hospital care that represented the first wave of COVID–19 in March 2020 in Spain led to a reduction in cases, which was accompanied by an increase in the risk profile of CABG patients but not PCI. On the other hand, the risk profile of both coronary revascularization procedures began before the pandemic, showing a significant temporal trend toward an increase in the risk profile. Further efforts should be aimed at studying and validating our results and evaluating other databases, regions, or countries.

## Figures and Tables

**Table 1:** Summary of CABG and PCI ordinary least squares models with included dummy variables for months.

	CABG				PCI			
	Estimate	Std. Error	t-Value	Pr(>|t|)	Estimate	Std. Error	t-Value	Pr(>|t|)
β_0_	2.890 × 10^−2^	6.733 × 10^−4^	42.930	<2 × 10^−16^	2.569 × 10^−2^	6.948 × 10^−4^	36.979	< 2 × 10^−16^
β_1_	6.870 × 10^−5^	1.178 × 10^−5^	5.830	5.58 × 10^−7^	1.072 × 10^−4^	1.216 × 10^−5^	8.818	2.29 × 10^−11^
β_2_	2.512 × 10^−3^	1.056 × 10^−3^	2.379	0.02164	1.747 × 10^−3^	1.089 × 10^−3^	1.604	0.115772
β_3_	2.983 × 10^−4^	1.768 × 10^−4^	1.687	0.09843	2.527 × 10^−4^	1.824 × 10^−4^	1.385	0.172802
Jan	9.700 × 10^−4^	7.948 × 10^−4^	1.220	0.22869	1.637 × 10^−3^	8.203 × 10^−4^	1.995	0.052087
Feb	−1.034 × 10^−3^	7.940 × 10^−4^	−1.302	0.19946	−8.006 × 10^−4^	8.194 × 10^−4^	−0.977	0.333804
Mar	−1.306 × 10^−3^	7.934 × 10^−4^	−1.646	0.10672	−9.639 × 10^−4^	8.188 × 10^−4^	−1.177	0.245299
Apr	−1.346 × 10^−3^	8.300 × 10^−4^	−1.622	0.11186	−1.053 × 10^−3^	8.565 × 10^−4^	−1.229	0.225484
May	−2.719 × 10^−3^	8.173 × 10^−4^	−3.326	0.00176	−2.370 × 10^−3^	8.435 × 10^−4^	−2.810	0.007304
Jun	−2.767 × 10^−3^	8.062 × 10^−4^	−3.432	0.00129	−2.426 × 10^−3^	8.320 × 10^−4^	−2.916	0.005509
Jul	−2.471 × 10^−3^	7.967 × 10^−4^	−3.102	0.00332	−2.109 × 10^−3^	8.221 × 10^−4^	−2.565	0.013709
Aug	−3.403 × 10^−3^	7.888 × 10^−4^	−4.315	8.64 × 10^−5^	−2.930 × 10^−3^	8.140 × 10^−4^	−3.600	0.000789
Sep	−3.558 × 10^−3^	7.826 × 10^−4^	−4.547	4.09 × 10^−5^	−3.254 × 10^−3^	8.076 × 10^−4^	−4.030	0.000213
Oct	−2.686 × 10^−3^	7.781 × 10^−4^	−3.452	0.00122	−2.407 × 10^−3^	8.030 × 10^−4^	−2.998	0.004418
Nov	−1.920 × 10^−3^	7.755 × 10^−4^	−2.476	0.01710	−2.354 × 10^−3^	8.003 × 10^−4^	−2.941	0.005148

**Table 2:** Wald test.

	CABG				PCI			
	Res.df	Df	F	Pr(>F)	Res.df	Df	F	Pr(>F)
Unrestricted model	45				45			
Restricted model	56	−11	7.4792	4.204 × 10^−7^	56	−11	7.5794	3.531 × 10^−7^

## Data Availability

The data used to support the findings of the present study are available from the corresponding author upon request.

## Supplementary Materials

The following supporting information can be downloaded at: https://www.mdpi.com/article/10.3390/jcdd10030102/s1, Tables S1 and S2: Main characteristics of the patients receiving coronary revascularizations.
