# Supplementary material for: The Effect of Coronavirus Outbreak on the Utilization of Coronary Revascularization Procedures: An Interrupted Time Series Analysis
Source: J Cardiovasc Dev Dis. 2023 Feb 27;10(3):102. doi: 10.3390/jcdd10030102 (PMC10055742; doi:10.3390/jcdd10030102)
Supplement: Supplementary file 1 [file jcdd-10-00102-s001.zip › jcdd-2150071-supplementary.pdf]

Table 1. Main characteristics of the patients receiving coronary artery by-pass surgery (CABG) during the time-period analyzed

YEAR	MONTH	Women (%)	Admission to ICU (%)	Elixhauser Comorbidity Index*	Age*	Severity*	Risk of Mortality*
2016	1.00	23,90	31,16	2,02	67,70	2,16	1,98
	2.00	23,58	30,28	2,08	67,95	2,10	1,94
	3.00	23,62	30,18	2,01	67,35	2,14	1,95
	4.00	22,81	29,56	2,06	67,14	2,06	1,92
	5.00	22,16	30,55	2,01	67,15	2,03	1,84
	6.00	23,02	30,60	2,17	67,73	2,08	1,89
	7.00	22,47	31,25	2,04	66,68	2,08	1,86
	8.00	23,08	31,78	1,97	67,09	2,11	1,91
	9.00	22,08	29,03	2,03	66,41	2,05	1,86
	10.00	24,43	31,72	2,05	67,83	2,05	1,94
	11.00	24,87	30,52	2,09	68,09	2,04	1,84
	12.00	22,52	32,04	2,08	67,65	2,17	1,97
2017	1.00	22,97	29,29	2,29	67,40	2,19	1,98
	2.00	23,10	32,30	2,20	68,93	2,16	1,94
	3.00	23,31	32,18	2,28	67,50	2,16	1,95
	4.00	23,91	32,05	2,25	67,17	2,17	2,01
	5.00	22,49	28,93	2,15	67,14	2,11	1,92
	6.00	24,77	31,10	2,24	67,89	2,15	1,99
	7.00	24,03	32,46	2,10	67,46	2,11	1,92
	8.00	23,50	31,32	2,16	67,45	2,11	1,90
	9.00	23,36	31,26	2,15	67,85	2,14	1,94
	10.00	24,58	30,37	2,15	67,29	2,16	1,94
	11.00	23,89	33,96	2,22	67,43	2,14	1,98
	12.00	23,65	31,33	2,19	68,17	2,20	2,06
2018	1.00	23,40	35,87	2,22	67,67	2,23	1,99
	2.00	23,45	33,53	2,30	67,44	2,23	2,02
	3.00	24,49	33,27	2,32	67,26	2,25	1,95
	4.00	22,77	33,93	2,24	67,11	2,16	1,93
	5.00	22,18	35,29	2,24	68,10	2,19	1,93
	6.00	24,06	36,82	2,21	67,22	2,21	1,96
	7.00	23,56	35,99	2,02	66,14	2,14	1,92
	8.00	24,66	34,14	1,99	66,71	2,13	1,84
	9.00	24,45	36,47	2,02	66,53	2,07	1,78
	10.00	24,54	37,31	1,99	68,01	2,07	1,81
	11.00	23,67	35,02	2,23	67,29	2,18	1,92
	12.00	23,44	36,24	2,08	66,96	2,16	1,93
2019	1.00	23,14	37,63	2,22	67,55	2,24	1,97
	2.00	25,06	35,60	2,09	67,19	2,13	1,88
	3.00	24,11	35,17	2,04	67,38	2,13	1,88

	4.00	23,17	34,95	2,09	67,28	2,20	1,95
	5.00	23,91	38,99	2,12	67,60	2,21	1,93
	6.00	25,12	37,03	2,01	67,39	2,10	1,83
	7.00	26,21	35,86	2,06	67,93	2,18	1,89
	8.00	26,43	36,12	2,05	66,93	2,20	1,94
	9.00	24,32	37,12	2,24	66,92	2,26	1,98
	10.00	24,47	36,11	2,22	67,58	2,23	1,99
	11.00	24,52	37,20	2,13	67,70	2,28	1,97
	12.00	22,41	33,97	2,15	67,39	2,25	2,00
2020	1.00	23,66	36,40	2,21	67,72	2,29	2,04
	2.00	23,13	35,20	2,07	67,81	2,19	1,94
	3.00	23,12	37,74	2,16	67,03	2,23	1,94
	4.00	24,04	37,47	2,17	65,05	2,09	1,92
	5.00	23,73	33,63	2,09	65,99	2,12	1,86
	6.00	25,40	32,13	2,25	66,69	2,25	1,99
	7.00	24,77	30,56	2,13	67,83	2,19	1,93
	8.00	24,20	32,95	2,16	67,21	2,20	1,92
	9.00	23,87	35,07	2,19	66,62	2,21	1,94
	10.00	23,97	33,73	2,23	66,57	2,25	2,05
	11.00	0,00	0,00	2,19	67,24	2,23	1,96
	12.00	0,00	0,00	2,29	66,80	2,35	2,09

ICU: Intensive Care Unit

* means

Table 2. Main characteristics of the patients receiving percutaneous coronary interventions (PCI) during the time-period analyzed

**Year**	**Month**	Women	Admission to ICU (%)	Elixhauser Comorbidity Index*	Age*	Severity*	Risk of Mortality*
2016	1.00	23,90	31,16	1,49	65,76	1,69	1,59
	2.00	23,58	30,28	1,48	65,78	1,68	1,60
	3.00	23,62	30,18	1,50	65,65	1,70	1,62
	4.00	22,81	29,56	1,48	65,27	1,69	1,58
	5.00	22,16	30,55	1,47	65,62	1,66	1,57
	6.00	23,02	30,60	1,49	65,06	1,66	1,57
	7.00	22,47	31,25	1,53	65,24	1,67	1,56
	8.00	23,08	31,78	1,46	64,89	1,69	1,59
	9.00	22,08	29,03	1,47	65,39	1,67	1,56
	10.00	24,43	31,72	1,47	66,09	1,67	1,57
	11.00	24,87	30,52	1,55	66,41	1,68	1,58
	12.00	22,52	32,04	1,51	66,47	1,71	1,61
2017	1.00	22,97	29,29	1,52	65,99	1,70	1,61
	2.00	23,10	32,30	1,48	65,71	1,65	1,54
	3.00	23,31	32,18	1,54	65,41	1,66	1,57
	4.00	23,91	32,05	1,52	65,22	1,69	1,60
	5.00	22,49	28,93	1,48	65,59	1,68	1,57
	6.00	24,77	31,10	1,47	65,29	1,63	1,53
	7.00	24,03	32,46	1,52	65,52	1,67	1,58
	8.00	23,50	31,32	1,49	65,17	1,64	1,56
	9.00	23,36	31,26	1,52	65,55	1,67	1,57
	10.00	24,58	30,37	1,48	65,19	1,66	1,58
	11.00	23,89	33,96	1,57	66,27	1,70	1,60
	12.00	23,65	31,33	1,57	65,92	1,70	1,63
2018	1.00	23,40	35,87	1,48	65,67	1,70	1,62
	2.00	23,45	33,53	1,54	65,81	1,72	1,62
	3.00	24,49	33,27	1,54	65,63	1,71	1,61
	4.00	22,77	33,93	1,58	65,42	1,70	1,60
	5.00	22,18	35,29	1,51	65,22	1,67	1,58
	6.00	24,06	36,82	1,56	65,54	1,69	1,59
	7.00	23,56	35,99	1,53	64,86	1,67	1,57
	8.00	24,66	34,14	1,54	64,91	1,67	1,58
	9.00	24,45	36,47	1,58	65,57	1,70	1,61
	10.00	24,54	37,31	1,55	66,09	1,69	1,60
	11.00	23,67	35,02	1,56	65,91	1,70	1,61
	12.00	23,44	36,24	1,61	66,02	1,75	1,67
2019	1.00	23,14	37,63	1,57	66,20	1,72	1,63
	2.00	25,06	35,60	1,57	65,59	1,71	1,62
	3.00	24,11	35,17	1,55	65,71	1,71	1,61

	4.00	23,17	34,95	1,59	66,14	1,72	1,62
	5.00	23,91	38,99	1,57	66,04	1,69	1,59
	6.00	25,12	37,03	1,60	65,62	1,69	1,61
	7.00	26,21	35,86	1,61	65,53	1,67	1,58
	8.00	26,43	36,12	1,55	65,35	1,69	1,60
	9.00	24,32	37,12	1,61	65,86	1,69	1,61
	10.00	24,47	36,11	1,61	66,31	1,69	1,60
	11.00	24,52	37,20	1,63	66,49	1,71	1,61
	12.00	22,41	33,97	1,60	66,04	1,73	1,64
2020	1.00	23,66	36,40	1,58	65,89	1,73	1,64
	2.00	23,13	35,20	1,60	66,09	1,72	1,63
	3.00	23,12	37,74	1,55	65,50	1,71	1,62
	4.00	24,04	37,47	1,66	64,76	1,77	1,68
	5.00	23,73	33,63	1,65	64,76	1,75	1,63
	6.00	25,40	32,13	1,65	65,79	1,76	1,66
	7.00	24,77	30,56	1,69	65,44	1,76	1,65
	8.00	24,20	32,95	1,63	65,44	1,76	1,66
	9.00	23,87	35,07	1,64	65,73	1,75	1,63
	10.00	23,97	33,73	1,67	65,98	1,77	1,67
	11.00	0,00	0,00	1,66	65,98	1,76	1,66
	12.00	0,00	0,00	1,68	65,67	1,79	1,69

ICU: Intensive Care Unit

* means
